# How to Measure the Relative Length of the Lower Limbs

**Published:** 1878-06

**Authors:** Henry Banga

**Affiliations:** Chicago


### HOW TO MEASURE THE RELATIVE LENGTH OF THE LOWER LIMBS.

*Messrs. Editors:*

In the last issue of this *Journal*, Dr. Bartlett described an apparatus for measuring the relative length of the lower extremities, which reminds me of another method, for the same purpose, that recommends itself by its simplicity and accuracy.

The anterior superior spinous process of the ilium is carefully marked, on both sides with ink or a colored pencil, while the patient keeps a horizontal position in bed. He then, is directed to rise to his feet, and to assume as straight and natural a position as possible. Especially must both feet be brought into natural abduction, the heels touching, if possible, one the other. The practitioner who is standing at a distance of from two to three yards before the patient, will at once recognize the shorter leg from the lower position of the corresponding spinous process. An assistant should then put pieces of wood, one-eighth inch thick, under the foot of the shortened leg till the anterior superior spinous processes are on a level. The height of the wood block necessary to raise the lowered spinous process to the horizontal line, designates the accurate amount of shortening.

If the practitioner should not trust his eyes in judging of the symmetry of the pelvis, he can establish a very simple level by placing between the patient and himself a table whose edge will guide him in appreciating the horizontal relation of the marked processes, or he may resort to the instruments that stonecutters use in similar cases.

I hardly need add that the measure of the height of the pile of boards on which the shortened leg rests, should be taken while the patient is standing on it.

I am satisfied that this simple method of measuring the shortening of the limbs, enables us to demonstrate very small differences, such as require no correction at all. Furthermore, if the measure is taken while the patient is erect, the full weight of his body forces the muscles and joints of the lower extremities to assume their natural position. We should thus be able to avoid the many errors to which Dr. Bartlett has called attention, as resulting from differences in adduction, abduction and flexion.

Altogether I regard it as a matter of some importance that the profession should agree upon a uniform method of measuring the shortening of the lower limbs. In cases of fracture of the femur, for instance, the question remains still unsettled, whether preference should be given to the use of Hodge's splint, the double inclined plane, the plaster-of-Paris bandage, or the weight and pulley. Each of these apparatuses has its advocate, yet a final decision as to the *real* results obtained by each one, may only be expected, when, after the union of the fractured bone the relative length of both limbs is ascertained *in every case in the same manner.* I trust that the method described above, if fairly tested, will be found worthy of general adoption.

Respectfully,

HENRY BANGA, M. D.

CHICAGO, May 20, 1878.

---

TREATMENT OF HERNIA.—The writer publishes four new cases of hernia, treated by his method of injection of 70 per cent. alcohol. After complete reduction of the hernia, he injects into the subcutaneous tissue around the hernial canal, the contents of one or two hypodermic syringes, and repeats this in the course of a few days. The canal is thus gradually surrounded by a ring of induration. Suppuration is a *desirable* result of the injection. During the treatment the patient can walk about with a truss. Of four cases of large hernia, the canal was obliterated in two and considerably narrowed in the other cases.—C. Schwalbe (*Deutche Med. Wochenschr;* 1877. No. 45).

ANOTHER ECTROTIC IN SMALL-POX.—The powder consisting of four parts sulphur and precipitate, employed by Semaria with such success in eczema and acne, will, he now claims, prevent the unsightly cicatrization after variola. The suppurating pustules are to be first penciled with glycerine, and the powder afterward thickly strewed over them. The crust thus formed is cast off without leaving behind any cicatrices.—*Gazette Med. Ital. Lomb.*

